# Severe hypotension but not systemic inflammation or endothelial activation predicts encephalopathy in circulatory shock

**DOI:** 10.1016/j.aicoj.2026.100033

**Published:** 2026-02-18

**Authors:** Duc Nam Nguyen, Luc Huyghens, Truc Mai Nguyen, Johan Schiettecatte, Marc Diltoer, Wilfried Cools, Helene De Cuyper, David Rhapsorski, Johan Smitz, Haibo Zhang

**Affiliations:** a Department of Critical Care Medicine, Universitair Ziekenhuis Brussel, Vrije Universiteit Brussel, Brussels, Belgium; b Brain Resuscitation in Neurosciences Research Group, Faculty of Medicine, Vrije Universiteit Brussel, Brussels, Belgium; c Department of Cardiology, University Hospital Vaudois, Lausanne, Switzerland; d Department of Immunochemistry, Universitair Ziekenhuis Brussel, Vrije Universiteit Brussel, Brussels, Belgium; e Department of Biostatistics, Vrije Universiteit Brussel, Brussels, Belgium; f Keenan Research Centre for Biomedical Science of St. Michael’s Hospital, Unity Health Toronto, Department of Anesthesiology and Pain Medicine, Department of Physiology, and Interdepartmental Division of Critical Care Medicine, University of Toronto, Ontario, Canada

**Keywords:** Encephalopathy, Circulatory shock, S100B protein, Hypotension, Inflammation, Endothelial activation

## Abstract

**Background:**

Encephalopathy is a frequent complication of circulatory shock and is associated with adverse outcomes. Whether encephalopathy is driven primarily by systemic inflammation, endothelial activation or cerebral hypoperfusion remains uncertain.

**Methods:**

We retrospectively studied 198 intensive care unit (ICU) patients with circulatory shock (95 septic shock, 103 non-septic shock). Encephalopathy (coma and delirium) was assessed using the Glasgow Coma Scale, Richmond Agitation-Sedation Scale, and Confusion Assessment Method for the ICU. Neuroinflammation or blood-brain barrier (BBB) dysfunction was evaluated using serum S100B protein. Systemic inflammation and endothelial activation were assessed using serum C-reactive protein (CRP), Matrix metalloproteinase-9 (MMP-9), Intercellular Adhesion Molecule -1 (ICAM-1) and Vascular Endothelial Growth Factor (VEGF). Severe hypotension was defined a priori as mean arterial pressure (MAP) <50 mmHg sustained ≥1 min; we also quantified the number of episodes and cumulative duration of MAP <60 and <50 mmHg across the first 72 h. Multivariable logistic regression and mixed-effect models examined associations with encephalopathy and ICU outcomes.

**Results:**

Encephalopathy developed in 140 patients (71%): 31 (23%) with coma and 99 (71%) with delirium. Severe hypotension (OR: 2.56 (1.18, 4.75), p = 0.022), longer sedation duration (OR: 1.09 (1.02, 1.18), p = 0.017), ICU-acquired infections (OR: 1.61(0.73, 3.54), p = 0.021), and elevated S100B (OR: 1.72 (0.66, 3.65), p = 0.03) were associated with encephalopathy. In contrast, systemic inflammation (CRP, MMP-9) and endothelial activation (ICAM-1, VEGF) were not associated with encephalopathy. Despite higher systemic inflammation in septic shock, the prevalence of encephalopathy and structural brain injury was similar to non-septic shock.

**Conclusions:**

In circulatory shock, encephalopathy is most strongly associated with recurrent/severe hypotension (MAP <50 mmHg) and markers of neuroinflammation, not systemic inflammation or endothelial activation.

## Graphical abstract

**Figure:**
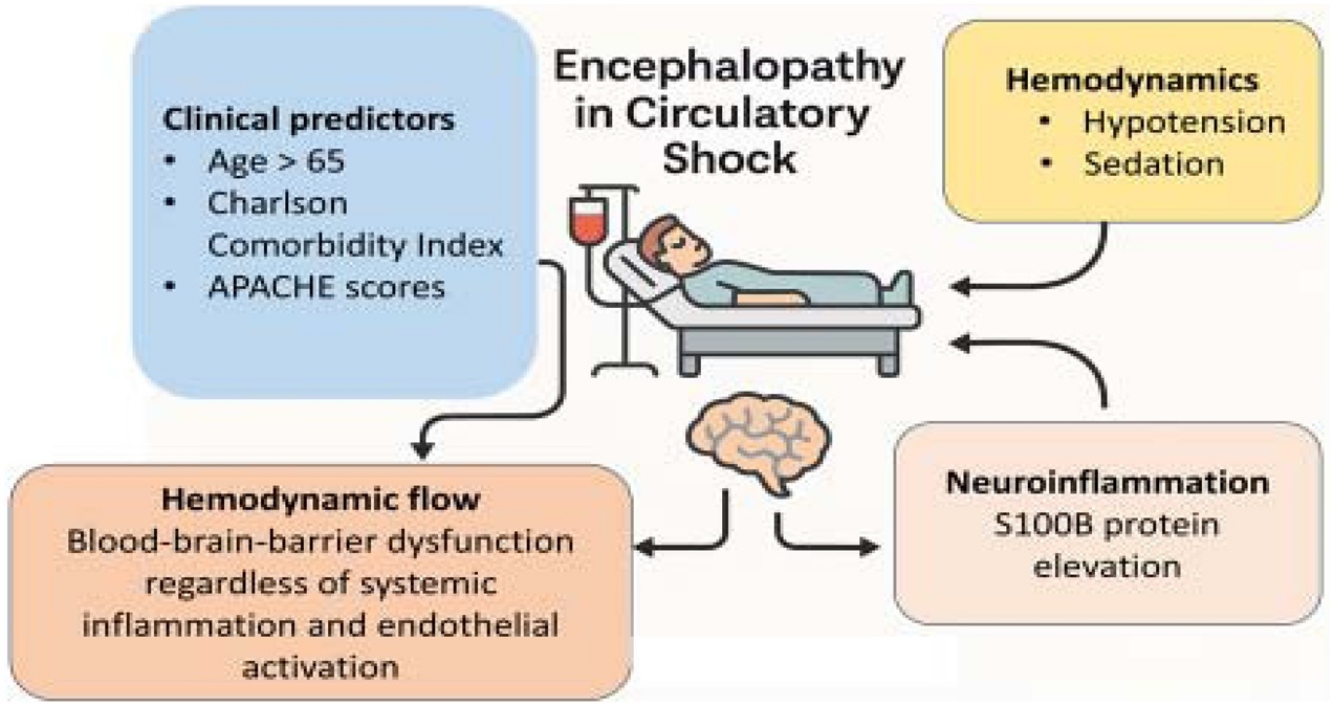

## Introduction

Encephalopathy, manifesting as delirium or coma, is common in patients with circulatory shock and is associated with increased morbidity and mortality in the intensive care unit (ICU) [1,2]. Its pathogenesis is multifactorial, involving cerebral hypoperfusion, blood-brain-barrier (BBB) dysfunction, and neuroinflammation triggered by systemic insults. Proposed mechanisms include: (1) Cerebral hypoperfusion due to hypotension, ischemia, or impaired autoregulation [3,4]. (2) Systemic and endothelial inflammatory responses to circulating pathogen-associated and damage-associated molecular patterns (PAMPs and DAMPs) that can compromise BBB integrity [5]. (3) Secondary neuroinflammation, driven by activation of astrocytes and microglia and by influx of neurotoxic metabolites such as cytokines, glutamate, immune cells, and reactive oxygen species [[6], [7], [8]]. Prior studies suggest that both delirium and systemic inflammation are prevalent in septic and non-septic shock [[5], [6], [7],[9], [10], [11], [12]]. However, the relationship between encephalopathy and systemic inflammation remains unclear, particularly in non-septic shock (cardiogenic, hypovolemic, or obstructive). Existing literature is limited by heterogeneous patient populations, inclusion of primary neurological disorders, and variable timing of biomarker assessment [[13], [14], [15]]. It also remains uncertain whether systemic inflammation synergizes with hypotension to worsen encephalopathy, or whether encephalopathy is largely driven by cerebral hypoperfusion independent of systemic inflammatory response.

To address knowledge gaps, we conducted a retrospective study in a well-characterized cohort of ICU patients with septic and non-septic circulatory shock to determine: (1) Whether encephalopathy is associated with systemic inflammation or endothelial activation, as reflected by serum CRP, MMP-9, ICAM-1, and VEGF; These biomarkers may have a neurotoxic effect, impair the BBB integrity, and may worsen the outcome in sepsis and stroke patients. [[16], [17], [18], [19], [20], [21], [22], [23], [24]]. (2) Whether severe hypotension and shock etiology influence the development of encephalopathy and its associated ICU outcomes. We hypothesized that severe hypotension would be the dominant predictor of encephalopathy, whereas systemic inflammatory and endothelial activation biomarkers would be less predictive.

Neuroinflammation and/or the BBB dysfunction was assessed by S100B protein which is released from activated astrocytes through a damaged BBB into the serum [[25], [26], [27]].

## Patients and methods

### Study design and patient population

We conducted a retrospective observational study in a 24-bed mixed medical-surgical ICU at Universitair Ziekenhuis Brussel, Belgium. All adult patients (≥18 years) admitted with circulatory shock between February 2014 and January 2018 were screened. Eligible required vasopressor support to maintain mean arterial pressure (MAP) ≥65 mmHg after fluid resuscitation, lactate >2 mmol/L, presence of organ hypoperfusion with the Sequential Organ Failure Assessment (SOFA) score >2, and biomarker measurements within the first 72 h after ICU admission. Shock etiology was classified as septic shock (SS) (Sepsis-3; managed per the 2016 Surviving Sepsis Campaign) [28] and non-septic shock (NS) [cardiogenic (CS), hypovolemic (HS), obstructive (OS) shock; anaphylactic shock excluded due to low prevalence] (Table 1). CS was recorded when meeting Society for Cardiovascular Angiography and Intervention (SCAI) stages C–D, typically occurring after acute coronary syndrome, post-cardiotomy syndrome, or acute-on-chronic heart failure [29]. HS included cases due to massive hemorrhage (e.g., gastrointestinal bleeding, aortic dissection, postpartum hemorrhage) or severe fluid loss (e.g., pancreatitis, prolonged diarrhea); and OS was primarily attributed to massive pulmonary embolism.

**Table 1:** Baseline ICU characteristics and clinical parameters in patients with and without encephalopathy, and stratified by shock etiology (septic vs. non-septic shock).

	Total n = 198	Encephalopathy n = 140 (71%)	No encephalopathy n = 58 (29%)	p-value	Septic shock n = 95 (48%)	Non-Septic shock n = 103 (52%)	p-value
Characteristics							
Age (years)	71 (61, 78)	74 (63, 80)	67 (52, 75)	**< 0.01**	68 (58, 77)	73 (64, 78)	0.112
Females, n (%)	68 (34)	45 (32)	23 (39)	0.396	25 (26)	43 (42)	**0.032**
APACHE scores	78 (56, 103)	79 (56, 102)	75 (56, 103)	0.164	79 (56, 102)	75 (56, 103)	0.891
SOFA score at inclusion	7 (5, 9)	7 (5, 9)	6 (4, 9)	0.424	7 (5, 9)	7 (5, 8)	0.412
Charlson comorbidity index	5 (3, 6)	5 (4, 6)	4 (3, 5)	**< 0.01**	4 (3, 6)	5 (3, 6)	0.433
Co-morbidities, n (%)							
Diabetes	41 (21)	33 (24)	8 (14)	0.176	18 (19)	23 (22)	0.680
Smoker and alcohol abuse	57 (29)	41(29)	16 (28)	0.550	34 (36)	22 (21)	**< 0.01**
Chronic pulmonary disorder	58 (29)	45 (32)	13 (22)	0.231	33 (36)	25 (24)	0.144
Arterial hypertension	109 (55)	81 (59)	28 (48)	0.242	49 (52)	61 (59)	0.348
Coronary artery disease	95 (48)	65 (46)	30 (52)	0.601	38 (40)	57 (55)	**0.044**
History of brain disorders	57 (29)	47 (34)	10 (17)	**0.032**	27 (28)	30 (29)	0.996
History of sepsis	46 (23)	30 (21)	16 (28)	0.453	28 (30)	18 (17)	0.067
History of kidney injury	43 (22)	33 (24)	10 (17)	0.427	17 (18)	26 (25)	0.280
Clinical and biological parameters at inclusion							
Temperature (°C)	37.1 (36.5, 37.7)	37.1 (36.5, 37.6)	37.2 (36.4, 37.7)	0.599	37.3 (36.5, 37.2)	37 (36.3, 36.9)	0.993
Heart rate (beats/min)	106 (92, 121)	106 (91, 123)	109 (97, 121)	0.347	109 (94, 123)	109 (106, 121)	0.71
Lowest mean arterial pressure (mmHg)	60 (52, 69)	60 (51, 69)	62 (54, 67)	0.522	58 (50, 68)	62 (53, 69)	0.345
pH	7.31 (7.24, 7.37)	7.32 (7.25, 7.37)	7.30 (7.23, 7.37)	0.766	7.29 (7.21, 7.36)	7.32 (7.26, 7.38)	0.171
PaCO2 (mmHg)	44 (38,50)	44 (39, 50)	43 (37, 50)	0.401	45 (39, 52)	43 (38, 49)	0.194
PaO2/FiO2 ratio	157 (100, 222)	157 (101, 223)	159 (93, 210)	0.977	128 (85, 192)	173 (120, 282)	**< 0.01**
Lactate (mmol/L)	2 (1, 3.6)	2 (1.25, 3.7)	1.55 (1.2, 3.2)	0.151	1.8 (1.2, 3.5)	2.2 (1.3, 3.6)	0.464
Hemoglobin (g/dl)	9.4 (8.1, 11.5)	9.2 (7.9, 11.3)	9.9 (8.5, 11.6)	0.161	10.4 (8.8, 11.6)	9.5 (7.5, 11)	**< 0.01**
Platelets (10.6/L)	150 (92, 261)	149 (92, 246)	172(94, 264)	0.755	199 (125, 286)	123 (86, 209)	**< 0.01**
Urea (mg/dl)	56 (36, 81)	57 (37, 84)	45 (34, 76)	0.144	64 (37, 94)	48 (34, 75)	**0.035**
Creatinine (mg/dl)	1.26 (0.92, 1.8)	1.28 (0.97, 1.83)	1.2 (0.86, 1.70)	0.293	1.34 (0.95, 2.07)	1.22 (0.9, 1.54)	0.118
Dobutamine, n (%)	109 (53)	72 (51)	37 (64)	0.151	46 (48)	63 (61)	0.235
Dobutamine (μg/kg/min)	3 (0, 5)	2 (0, 5)	5 (0, 7)	0.06	1 (0, 5)	4 (0, 7)	0.105
Noradrenaline, n (%)	119 (60)	83 (59)	36 (62)	0.837	56 (59)	63 (61)	0.952
Noradrenaline (μg/kg/min)	0.07 (0, 0.16)	0.07 (0, 0.18)	0.07 (0, 0.15)	0.974	0.08 (0, 0.16)	0.06 (0, 0.15)	0.558
Remifentanil, n (%)	154 (77)	109 (81)	45 (78)	1.000	73 (77)	80 (78)	0.632
Remifentanil (μg/kg/min)	0.01 (0, 0.02)	0.04 (0, 0.03)	0.01 (0, 0.03)	0.244	0.01 (0, 0.03)	0.01 (0, 0.02)	0.536
Midazolam, n (%)	64 (32)	46 (33)	18 (31)	0.934	20 (21)	26 (25)	0.643
Midazolam (ml/min)	1.5 (0, 2.4)	1.5 (0, 2)	1.4 (0, 3)	0.858	3.3 (1, 6)	3.6 (1, 5.4)	0.952
Propofol, n (%)	155 (78)	110 (82)	45 (78)	1.000	75 (79)	80 (78)	0.391
Propofol (ml/min)	3.6 (1, 4.6)	3.6 (1, 5.4)	3.4 (1, 5.9)	0.964	1.5 (0, 2)	1.4 (0, 3)	0.177

The values were presented as median (interquartile) unless stated otherwise.

### Exclusion criteria

We excluded patients who recovered from hypotension within 24 h of ICU admission; died from refractory shock or had a Do-Not-Resuscitate order within 48 h; had major comorbidities (terminal malignancy, severe dementia, advanced neuromuscular disease, end-stage organ failure); had primary neurological conditions or extracerebral sources of S100B release (post-cardiac arrest, polytrauma, neurotrauma, meningitis/encephalitis, intracerebral/subarachnoid hemorrhage, stroke, morbid obesity or severe burns) [25]; were admitted for acute alcohol/drug intoxication; or had missing biomarker measurements within 72 h.

### ICU management protocols

Hemodynamic management targeted MAP ≥ 65 mmHg with crystalloids and norepinephrine, with adjunct agents as indicated [25]. Cardiogenic shock patients received inotropes/vasopressors and mechanical circulatory support (intra-aortic balloon pump [IABP], extracorporeal membrane oxygenation [ECMO]) when appropriate [30]. Hypovolemic shock was treated with fluid/transfusions and definitive hemostasis. Obstructive shock (e.g., pulmonary embolism) received thrombolysis and heparin as appropriate. Mechanical ventilation used a lung-protective strategy (tidal volumes 6−7 mL/kg predicted body weight, plateau pressure <30 cmH_2_O, driving pressure <15 cmH_2_O). Sedation and analgesia targeted Richmond Agitation Sedation Scale (RASS) −1 to −3 using propofol and/or midazolam with opioids (morphine or remifentanil).

### Definition of hypotension and encephalopathy

Hypotension was defined as MAP < 60 mmHg and severe hypotension as MAP < 50 mmHg (lower limit of cerebral autoregulation) sustained ≥1 min. MAP values were obtained from continuous invasive arterial blood pressure monitoring and documented in the ICU nursing charts. The number of hypotensive episodes and their cumulative duration were estimated for each patient over the first three days after ICU admission, corresponding to the period of biomarker sampling.

Encephalopathy comprised coma [Glasgow Coma Scale (GCS) <8 without sedation or RASS ≤ −4] or delirium [Confusion Assessment Method for ICU (CAM-ICU) positive; hyperactive RASS ≥ +1; hypoactive RASS ≤ −1]. Encephalopathy assessment was recorded at two time-points: at admission before sedation and after sedation withdrawal. Structural brain injury was adjudicated by computer tomography (CT scanner) and magnetic resonance imaging (MRI) when available.

### Biomarker measurements

At the first 24 h after ICU admission and daily during two consecutive days, we measured with the ELISA technique: S100B (neuroinflammation/BBB dysfunction), C-reactive protein (CRP) and Matrix metalloproteinase-9 (MMP-9) (systemic inflammation), Intercellular Adhesion Molecule -1 (ICAM-1) and Vascular Endothelial Growth Factor (VEGF) (endothelial activation). Routine cytokine assays (interleukins −1/−6/ tumor necrosis factor-alpha / interferon gamma) were not available during the study period.

### Data collection

We collected demographics, comorbidities (Charlson Comorbidity Index), APACHE III and SOFA (day 3), hemodynamics (lowest daily MAP; number of episodes/duration of MAP <60 and <50 mmHg; vasopressor duration), ICU therapies (mechanical ventilation duration, sedation duration, vasopressors, renal replacement therapy, ECMO, ICU-acquired infections), neurological assessments (GCS, RASS, CAM-ICU, coma, delirium subtype, structural brain injury), and ICU/hospital outcomes. The sedatives doses were available as ml/h although infusion pumps were prepared according to standard protocols (μg/kg/min or mg/kg/h).

### Study outcomes

Primary outcome: development of encephalopathy (coma and delirium) during ICU stay. Secondary outcomes: association of hypotension, systemic inflammation, and endothelial activation with encephalopathy; impact of shock etiology on encephalopathy and ICU outcomes.

### Statistical analysis

Analyses used R 4.12.1 (R foundation for statistical computing, Vienna, Austria). Categorical variables: chi-square/Fisher’s exact; continuous variables: Wilcoxon rank-sum test for non-normally distributed data. Correlations: Spearman. Biomarker associations: Linear mixed-effect models with patient-level random effects, adjusted for age, Charlson index, APACHE III, SOFA (day 3), sedation duration, ICU-acquired infections, and encephalopathy/hypotension. Risk factors for encephalopathy were assessed using multivariable logistic regression, including the number of episodes of MAP <60 mmHg and <50 mmHg, adjusted for the above covariates and shock etiology. ICU mortality: Cox model with similar covariates. Interactions were between hypotension metrics and biomarkers were tested. Model selection used Akaike Information Criterion.

## Results

### Patient characteristics

**Fig. 1:**
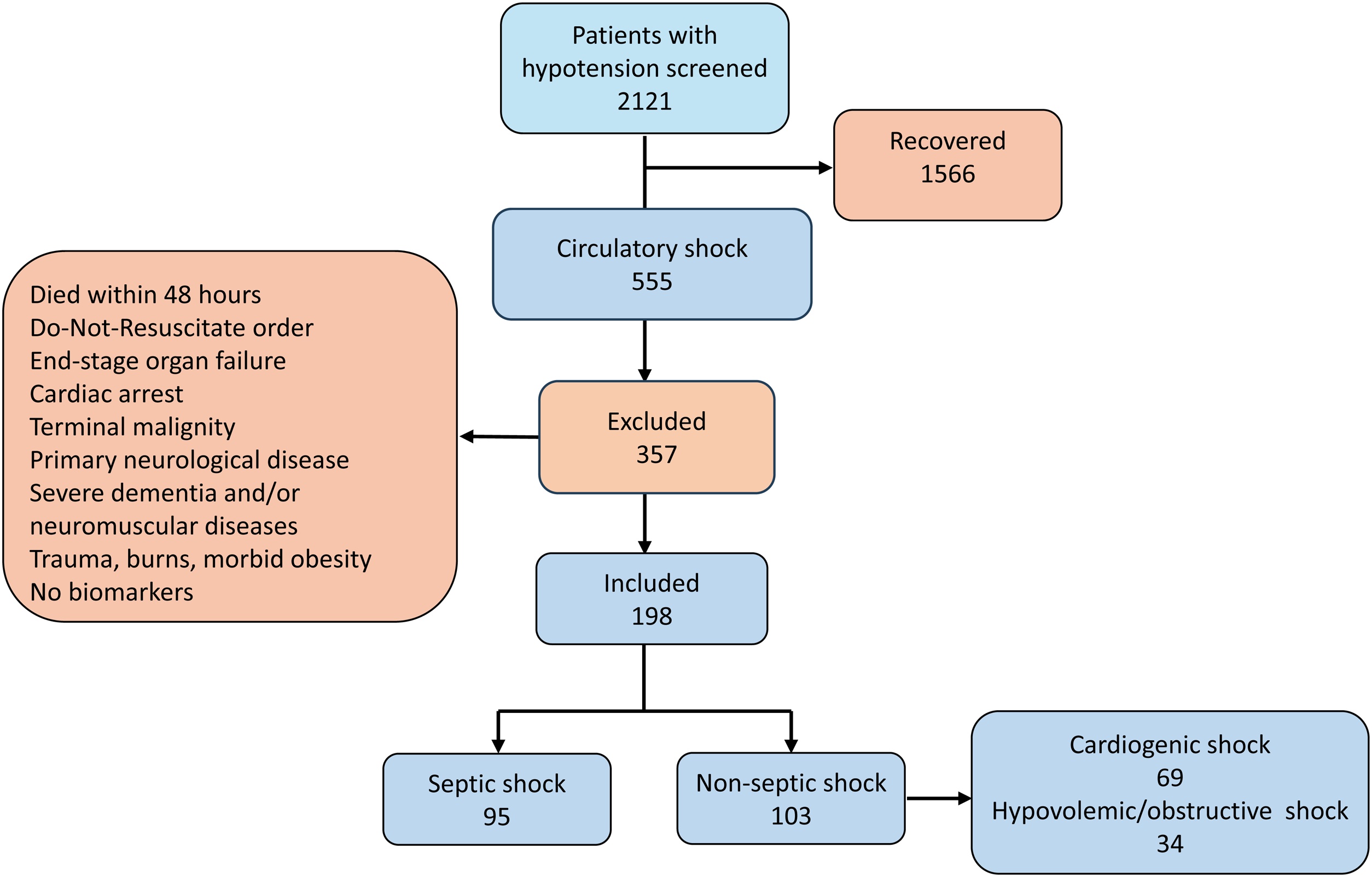
Flowchart of patients inclusion with circulatory shock.

Of 555 patients with circulatory shock, 198 met inclusion (95 septic shock; 103 non-septic) (Fig. 1). Median age 65 years; 43% female. Septic shock had fewer coronary risk factors and lower PaO₂/FiO₂, but higher hemoglobin/platelets. APACHE III, SOFA were similar across groups (Table 1).

### Incidence and subtypes of encephalopathy

Encephalopathy occurred in 140/198 (71%): coma 31 (23%), delirium 99 (71%); hyperactive 53 (53%), hypoactive: 46 (47%). (Supplementary Figure S1, Table 2).

**Table 2:** Neurological complications and ICU course in patients with and without encephalopathy, and stratified by shock etiology (septic vs. non-septic shock).

	Total n = 198	Encephalopathy n = 140 (71%)	No encephalopathy n = 58 (29%)	p-value	Septic shock n = 95 (48%)	Non-Septic shock n = 103 (52%)	p-value
Neurological complications							
Glasgow Coma Scale before sedation	15 (12,15)	14 (11, 15)	15 (15, 15)	**< 0.01**	14 (10,15)	15 (13,15)	**0.026**
Coma, n (%)	31 (16)	31 (22)	0	–	67 (73)	73 (69)	0.650
Delirium, n (%)	99 (51)	99 (71)	0	–	17 (18)	14 (13)	0.433
Delirium, days	2 (0, 5)	2 (0, 5)	0	–	46 (61)	53 (56)	0.808
Stroke, n (%)	10 (5)	10 (5)	0	–	4 (4)	6 (6)	0.409
Brain imaging results							
Number of patients underwent brain CT, n (%)	77 (39)	66 (47)	11 (19)	–	36 (38)	41 (40)	0.911
*Normal*	23 (30)	15 (11)	8 (57)	**< 0.01**	7 (7)	6 (6)	0.828
*Large Stroke, n (%)*	10 (5)	10 (5)	0 (0)	**0.036**	4 (4)	6 (6)	0.409
*Other brain lesions, n (%)*	44 (27)	41 (32)	3 (15)	**< 0.01**	25 (26)	29 (28)	0.933
*Atrophy, n (%)*	20 (10)	18 (13)	2 (3)	–	8 (8)	12 (10)	0.378
*Small hemorrhage, n (%)*	2 (1)	2 (1)	0 (0)	–	1 (1)	1 (1)	0.926
*Small ischemia, n (%)*	22 (11)	21 (15)	1 (2)	–	12 (13)	10 (9)	0.434
ICU evolution							
Mechanical ventilation, n (%)	186 (94)	134 (96)	52 (90)	0.090	86 (93)	100 (94)	0.998
Mechanical ventilation, days	6 (4, 16)	7 (4, 20)	5 (2, 9)	**< 0.01**	7 (4, 16)	6 (3, 17)	0.580
Sedation, n (%)	186 (94)	134 (96)	52 (90)	0.090	86 (93)	100 (94)	0.998
Sedation, days	5 (3, 10)	6 (4, 9)	4 (2, 5)	**< 0.01**	6 (4, 11)	5 (2, 9)	0.075
Vasopressors, n (%)	151 (76)	112 (80)	39 (67)	0.082	71 (75)	80 (78)	0.901
Vasopressors, days	4 (3, 5)	4 (2,4)	2 (0, 3)	**0.012**	3 (1, 5)	3 (1, 6)	0.760
ECMO or IABP, n (%)	59 (30)	38 (27)	21 (36)	0.297	5 (5)	54 (52)	**< 0.01**
Prevalence and duration of hypotension over the first three days.							
Episodes of mean arterial pressure < 50 mmHg over 3 study days, n	270	242	28	**< 0.01**	95	175	**0.012**
Mean arterial pressure < 50 mmHg duration (minutes)	5 (0, 30)	5 (0, 31)	0 (0, 0)	**< 0.01**	5 (0, 30)	5 (0, 30)	0.415
Episodes of mean arterial pressure < 60 mmHg over 3 study days, n	780	678	102	**< 0.01**	274	506	< **0.01**
Mean arterial pressure < 60 mmHg duration (minutes)	30 (0, 127)	50 (10,187)	30 (0,90)	**0.02**	40 (10,120)	30 (0,150)	0.847
SOFA score on day three	6 (3, 9)	7 (4, 9)	5 (3, 7)	**0.037**	6 (3, 9)	6 (3, 8)	0.954
ICU morbidities and mortality							
ICU mortality, n (%)	71 (36)	61 (44)	10 (17)	**< 0.01**	36 (38)	35 (34)	0.456
ICU stay, days	17 (7, 23)	14 (8, 26)	9 (5, 18)	**0.015**	13 (8, 20)	12 (6, 26)	0.960
In-hospital mortality after ICU, n (%)	14 (3)	12 (9)	2 (3)	0.098	7 (8)	7 (7)	0.874
In-hospital stay, days	27 (15, 44)	27 (15, 43)	24 (14,43)	0.458	26 (16, 43)	28 (13, 44)	0.951
ICU-acquired infection, n (%)	102 (51)	81 (58)	21 (36)	**< 0.01**	39 (41)	63 (61)	**< 0.01**
ICU-acquired infection after ICU admission, days	5 (0, 8)	4 (0, 5)	5 (0, 7)	0.054	5 (0, 4)	8 (0, 10)	**0.02**
Acute kidney injury, n (%)	72 (36)	51 (36)	21 (36)	1.000	37 (39)	35 (34)	0.560
Acute Respiratory distress syndrome, n (%)	20 (10)	18 (13)	2 (3)	0.081	11 (12)	9 (9)	0.669
Acute heart failure, n (%)	50 (25)	36 (26)	14 (24)	0.958	19 (20)	31 (30)	0.141

The values were presented as median (interquartile) unless stated otherwise.

IABP: intra-aortic balloon pump.

ECMO: extracorporeal membrane oxygenation.

Neuroimaging: Structural brain injury was identified in 44/140 imaged patients (31%). Patients with encephalopathy had more frequent large ischemic strokes (5% vs. 0%) and small hemorrhagic/ischemic lesions or atrophy (32% vs. 15 %) compared to those without encephalopathy (Table 2).

Timing: Among encephalopathy patients, 61% presented at ICU admission and 39% developed encephalopathy after sedation withdrawal. Admission encephalopathy was associated with more septic shock etiology, lower GCS, and higher S100B versus incident encephalopathy (Supplementary Table S1).

### Hemodynamics and hypotension

**Fig. 2:**
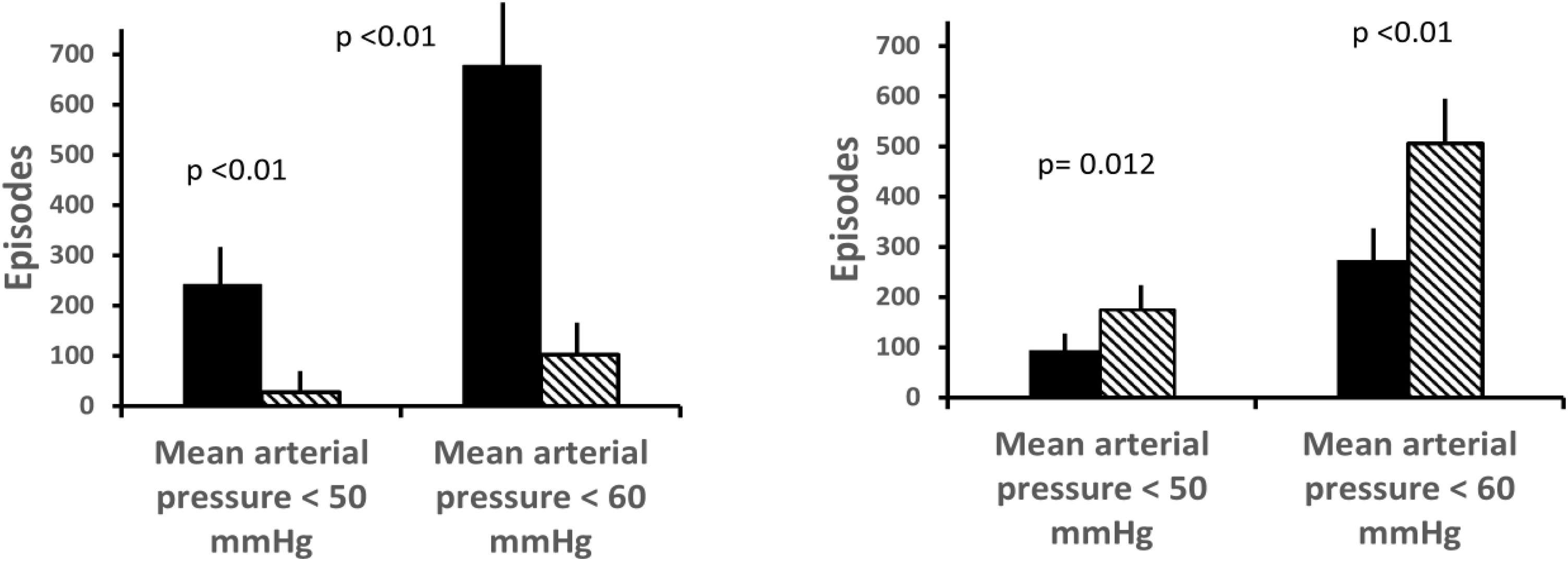
Prevalence of hypotension in patients with encephalopathy compared with those without (left), regardless of shock etiology (right).

**Fig. 3:**
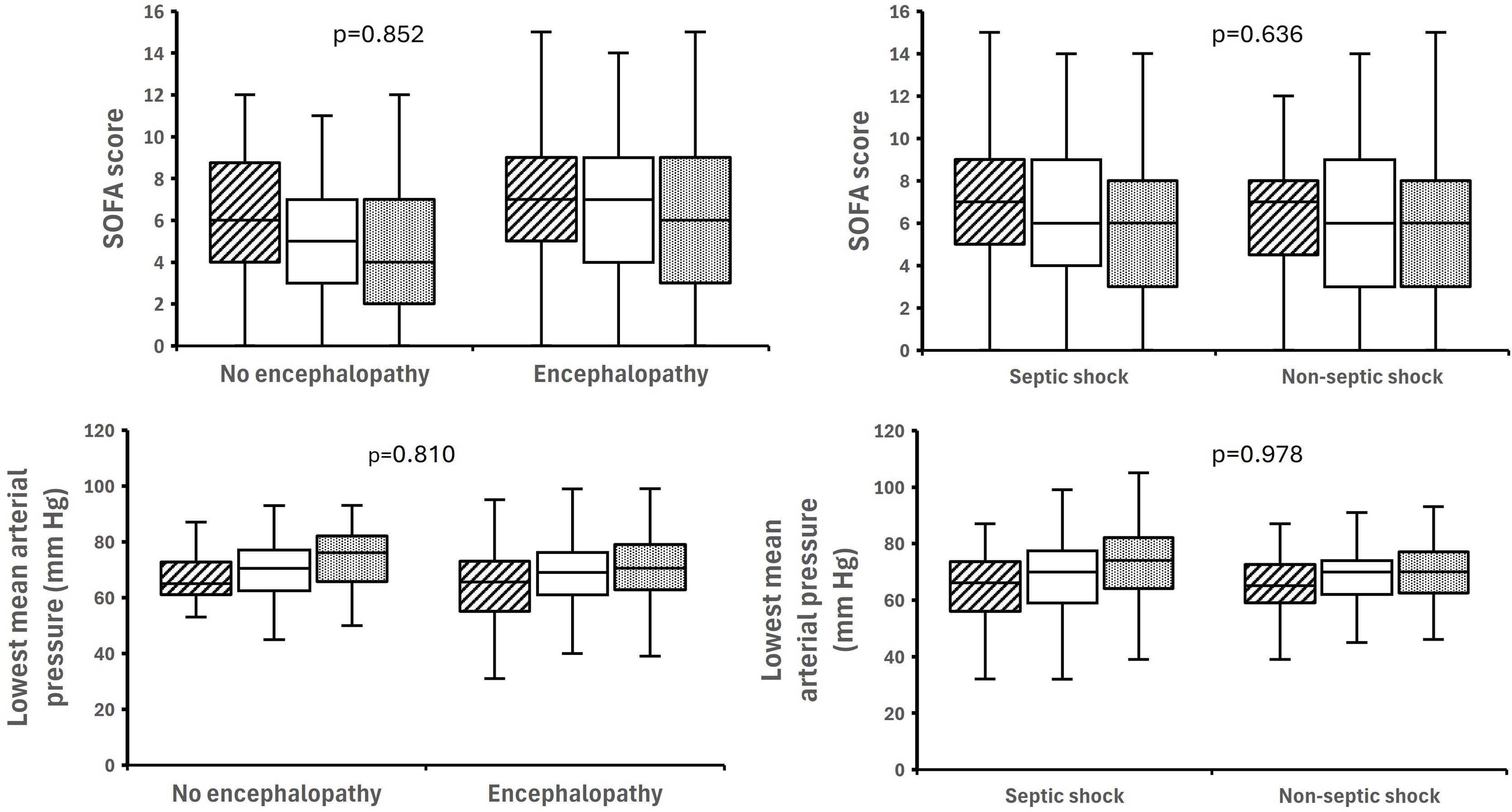
SOFA score and lowest mean arterial pressure in patients with circulatory shock. *Left panel*: Non-encephalopathy (n = 58) vs. encephalopathy (n = 140). *Right panel*: Non-septic shock (n = 95) vs. septic shock (n = 103*). P-values* reflect global between-group comparisons accounting for repeated measurements over three days.

Patients with encephalopathy had more episodes and longer cumulative duration of hypotension than those without (Fig. 2, Table 2) for similar SOFA and lowest MAP (Fig. 3). In adjusted models, repeated/severe hypotension (MAP <50 mmHg), by episode count was independently associated with encephalopathy (OR 2.56; 95% CI 1.18–4.75; p = 0.022) and not synergised with inflammation. Recurrent mild hypotension (MAP <60 mmHg) was not independently associated. Older patients (>65 years) were more prone to hypotension, structural brain injury, and encephalopathy (Supplementary Table S2, S3).

### Biomarkers of neuroinflammation, systemic inflammation, and endothelial activation

**Fig. 4:**
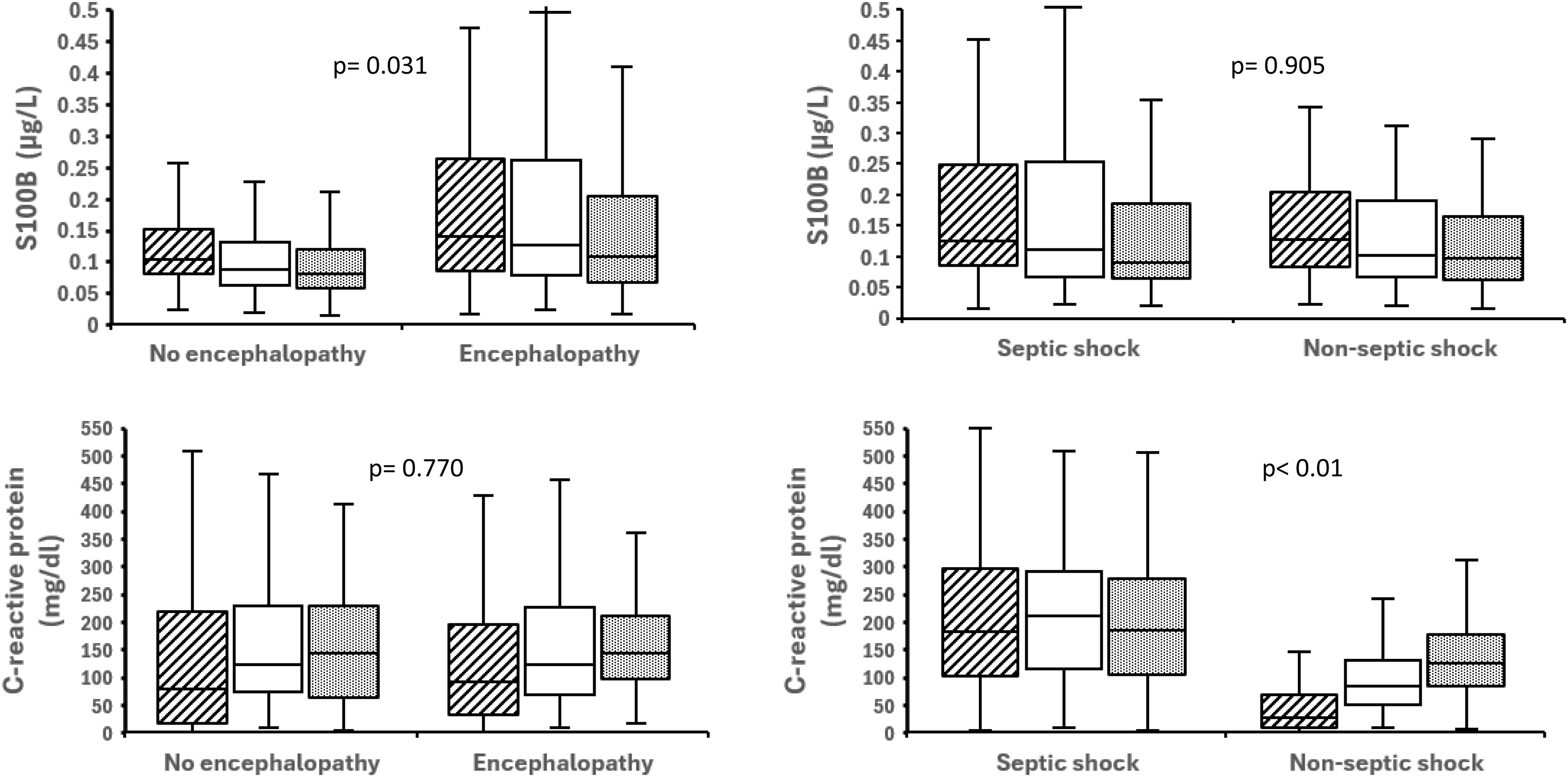
Serum S100B and C-reactive protein levels in patients with circulatory shock. *Left panel*: Non-encephalopathy (n = 58) vs. encephalopathy (n = 140). *Right panel*: Non-septic shock (n = 95) vs. septic shock (n = 103). *P-values* reflect global between-group comparisons accounting for repeated measurements over three days.

**Fig. 5:**
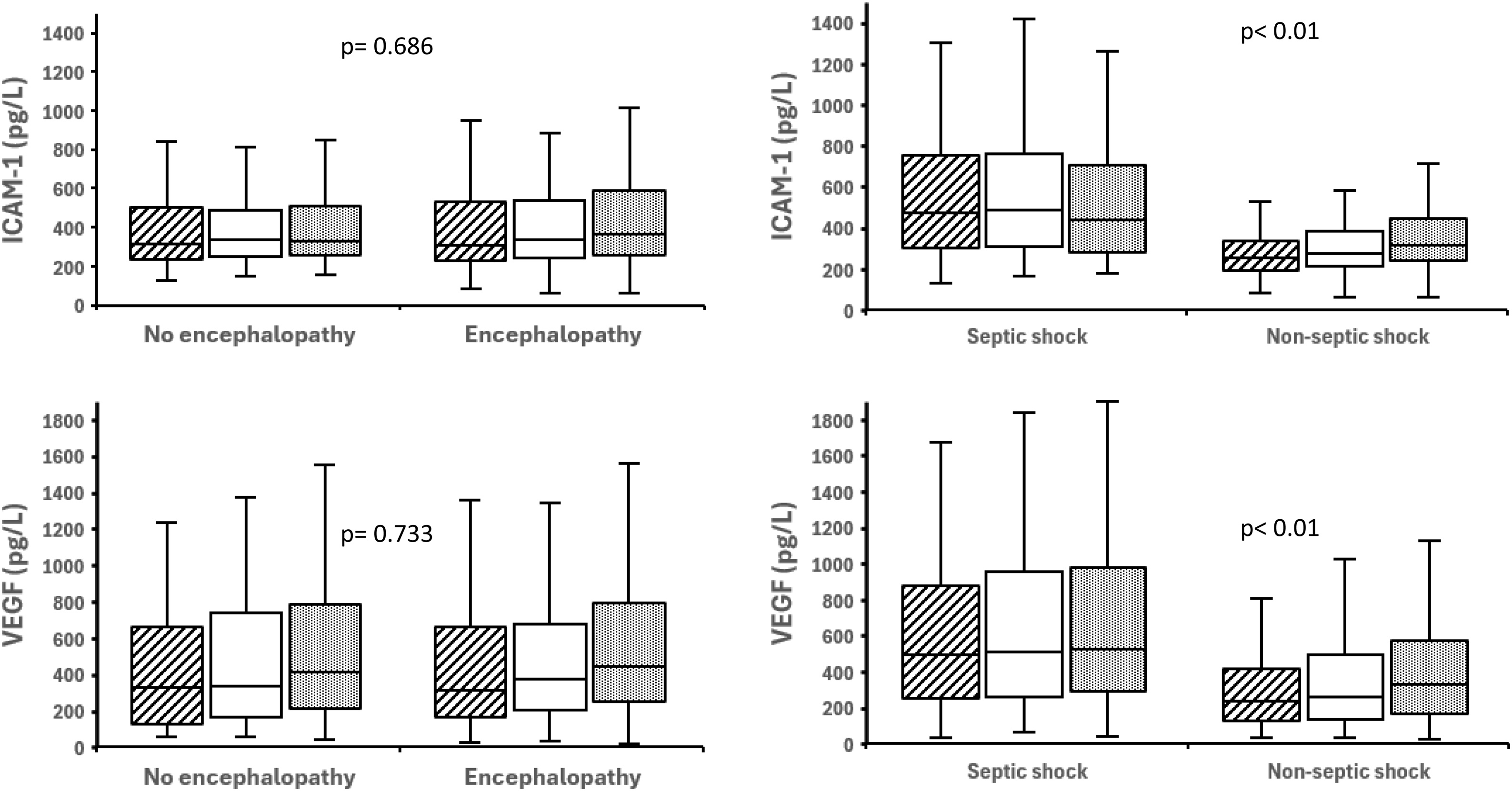
Serum ICAM-1 and VEGF levels in patients with circulatory shock. *Left panel*: Non-encephalopathy (n = 58) vs. encephalopathy (n = 140). *Right panel*: Non-septic shock (n = 95) vs. septic shock (n = 103). *P-values* reflect global between-group comparisons accounting for repeated measurements over three days.

S100B was higher with encephalopathy (p = 0.032), correlated with hypotensive episode counts (Spearman r = 0.45; p < 0.01) and associated with ICU-acquired infections (p = 0.028). Systemic inflammation (CRP, MMP-9) and endothelial activation (ICAM-1, VEGF) were not associated with encephalopathy (Fig. 4, Fig. 5, Table 3A). Septic shock had higher CRP, ICAM-1, VEGF than non-septic but similar S100B levels and encephalopathy (Table 3C).

**Table 3:** Serum biomarkers levels at inclusion in patients with circulatory shock: associations with encephalopathy, age, and shock etiology.

A: Associated with encephalopathy				
Serum biomarkers (normal values)	Total n = 198	Encephalopathy n = 140 (71%)	No encephalopathy n = 58 (29%)	p-value
S100B (< 0.105 μg/L)	0.13 (0.08, 0.23)	0.14 (0.09, 0.26)	0.10 (0.08, 0.15)	**0.013**
MMP-9 (< 21 ng/L)	98 (49, 160)	94 (52, 177)	74 (35, 177)	0.091
C-reactive protein (< 5 mg/dl)	89 (24, 197)	93 (31, 126)	79 (17, 218)	0.500
ICAM-1 (< 34 pg/L)	311 (226, 520)	310 (225, 528)	315 (237, 503)	0.811
VEGF (< 31 pg/L)	313 (149, 663)	311 (164, 662)	332 (128, 664)	0.698

B: Associated with age				
Serum biomarkers (normal values)	Total n = 198	AGE ≤ 65 n = 68 (34 %)	Age > 65 n = 130 (66 %)	p-value
S100B (< 0.105 μg/L)	0.13 (0.08, 0.23)	0.11 (0.080, 0.189)	0.13 (0.086, 0.243)	**0.03**
MMP-9 (< 21 ng/L)	98 (49, 160)	92 (50, 128)	99 (49, 164)	0.421
C-reactive protein (< 5 mg/dl)	89 (24, 197)	134 (36, 223)	78 (20, 179)	**0.044**
ICAM-1 (< 34 pg/L)	311 (226, 520)	428 (260, 695)	289 (214, 299)	**< 0.01**
VEGF (< 31 pg/L)	313 (149, 663)	377 (177, 691)	293 (144, 607)	0.200

C: Associated with shock etiology				
Serum biomarkers (normal values)	Total n = 198	Septic shock n = 95 (48%)	Non-Septic shock n = 103 (52%)	p-value
S100B (< 0.105 μg/L)	0.13 (0.08, 0.23)	0.13 (0.09, 0.26)	0.13 (0.08, 0.2)	0.899
MMP-9 (< 21 ng/L)	98 (49, 160)	105 (55, 188)	97 (43, 131)	0.110
C-reactive protein (5< mg/dl)	89 (24, 197)	183 (103, 296)	27 (10, 69)	**< 0.01**
ICAM-1 (< 34 pg/L)	312 (226, 520)	463 (292, 762)	258 (191, 347)	**< 0.01**
VEGF (< 31 pg/L)	313 (149, 663)	486 (253, 873)	242 (126, 428)	**< 0.01**

S100B: S100B protein.

MMP-9: Matrix metalloproteinase-9.

ICAM-1: Intercellular Adhesion Molecule-1.

VEGF: Vascular Endothelial Growth Factor.

Patients >65 years old had higher S100B, CRP, and ICAM-1 levels than younger patients (Table 3B, Supplementary Figures S2–S4). Biomarkers of inflammation and endothelial activation were weakly correlated (Supplementary Table S4).

### Impact of shock etiology

Encephalopathy prevalence and structural brain injury were similar between septic and non-septic shock (Table 2). Non-septic shock had more severe hypotension, greater use of IABP/ECMO, and more ICU-acquired infections.

### Risk factors for encephalopathy

**Fig. 6:**
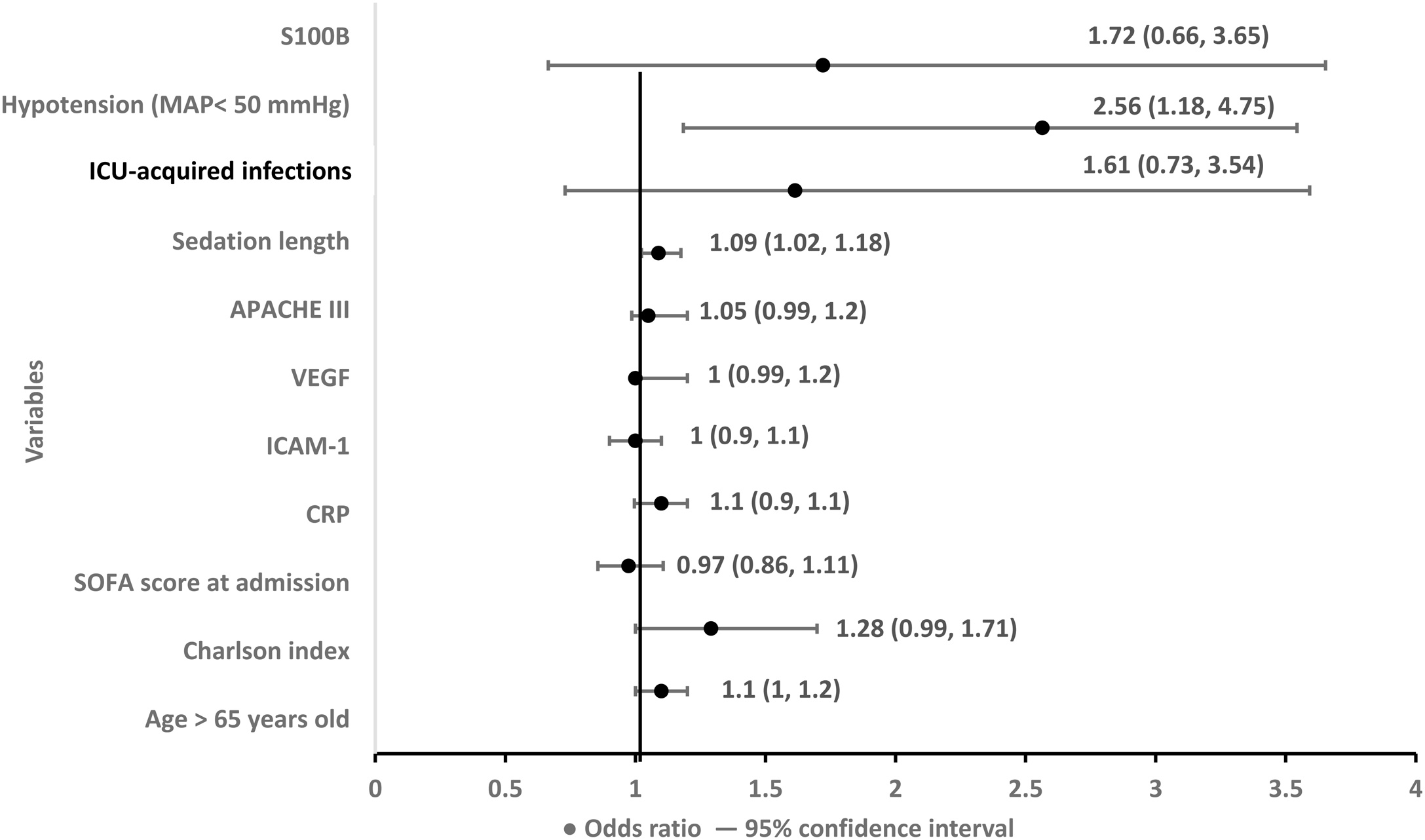
Risk factors for the development of encephalopathy in circulatory shock, expressed as odds ratio (95% confidence interval). Abbreviations: S100B, S100B protein; APACHE III, Acute Physiology and Chronic Health Evaluation III score; VEGF, Vascular Endothelial Growth Factor; ICAM-1, Intercellular Adhesion Molecule-1; CRP, C-reactive protein; SOFA, Sequential Organ Failure Assessment; Charlson index, the Charlton comorbidity index.

Independent factors (multivariable): severe hypotension (MAP < 50 mmHg), sedation duration, ICU-acquired infections, and higher Charlson Comorbidity Index were associated with encephalopathy. Systemic inflammatory and endothelial biomarkers were not independently predictors (Fig. 6).

### ICU and hospital outcomes

Overall ICU mortality was 43% (85/198), higher in encephalopathy (44% vs. 17%, p < 0.01). The in-hospital but not ICU mortality was higher in patients >65 years than <65 years old (Supplementary Table S3). After adjustment, encephalopathy itself was not an independent predictor of mortality; Charlson Comorbidity Index (OR 1.20; p = 0.024) and APACHE III scores (OR 1.01; p < 0.01) were significant.

## Discussion

In this retrospective study of 198 ICU patients with circulatory shock, we found that encephalopathy was common (71%) and associated with higher illness severity. The strongest independent risk factor was recurrent severe hypotension (MAP <50 mmHg), followed by longer sedation, ICU-acquired infections, and elevated S100B levels, a marker of neuroinflammation and blood-brain-barrier (BBB) dysfunction. In contrast, systemic inflammation (CRP, MMP-9) and endothelial activation (ICAM-1, VEGF) were not associated with encephalopathy, despite being elevated in septic shock. These findings collectively suggest that cerebral hypoperfusion and neuroinflammation, rather than systemic inflammatory activation, are the dominant link to of encephalopathy in circulatory shock. Moreover, hypotension and encephalopathy likely reflect components of global illness severity but not acting as independent determinants of outcome in shock states.

### Encephalopathy in circulatory shock: a perfusion-link complication

Our results reinforce the concept that encephalopathy in shock reflects a perfusion-link complication. Recurrent or prolonged MAP <50 mmHg episodes were consistently linked to encephalopathy and may associate with cerebral hypoperfusion, aligning with prior studies showing that cerebral autoregulation is frequently impaired in more than half of septic patients. Under such conditions, especially when a shift rightward the curve of autoregulation is present [33,34], transient drops below the autoregulatory threshold can result in regional cerebral hypoperfusion and ischemia, especially in vulnerable regions such as the hippocampus, cortex, and brainstem [[35], [36], [37], [38], [39]]. Prior studies show that MAP ≤50−55 mmHg may associated with delirium, coma, and in less arousal ICU patients and support this result [31,32]. While global cerebral blood flow may be preserved in sepsis [40,41], recent data using transcranial Doppler (TCD) and contrast-enhanced brain ultrasound show that microcirculation was altered and autoregulation is impaired in non-survivors [42]. The observed correlation between S100B elevation and number of MAP <50 mmHg episodes further may indicate that BBB dysfunction and astroglial activation may arise from hypotension-induced secondary regional microcirculatory ischemia rather than global hypoperfusion. In addition, previous brain disorders, comorbidities, longer sedation, impaired neurotransmission with age, all may aggravate encephalopathy in shock states [1,[3], [4], [5], [6],^43^]. Our results showed that elderly >65 years old who had higher S100B levels were prone to hypotension and more frequently affected by encephalopathy and structural brain injury on imaging than other patients. However, given the observational design and the absence of pre-ICU hemodynamic data, temporal precedence between hypotension and encephalopathy present at admission cannot be established. Associations identified in this study should therefore be interpreted as correlates of disease severity and vulnerability rather than evidence of causality.

### Systemic inflammation and endothelial activation: insufficient explanatory factors

Although inflammatory and endothelial activation are mechanistically linked to delirium in sepsis [[5], [6], [7], [8], [9], [10]], our study found no independent association between CRP, MMP-9, ICAM-1, or VEGF and encephalopathy in circulatory shock. This discrepancy may reflect several factors: (1) systemic biomarkers fluctuate over time and may not coincide with neuroinflammatory events; (2) their serum concentrations may not mirror cerebral compartment activity; and (3) the mechanisms of microcirculatory alterations may dominate over systemic inflammation, (4) specific biomarker for encephalopathy remains to be determined [[44], [45], [46], [47], [48]]. Moreover, markedly higher inflammatory biomarker levels in septic compared to non-septic shock, encephalopathy prevalence and structural brain injury rates were similar, suggesting that inflammation alone is neither necessary nor sufficient for encephalopathy in shock.

Our findings thus extend previous work by showing that hypotension and impaired regional cerebral perfusion, rather than inflammatory activation, are the proximate factors linking shock physiology to encephalopathy. This interpretation aligns with growing evidence that BBB dysfunction and neuroinflammation (reflected by S100B) can occur independently of cytokine surges [49,50].

### S100B as a surrogate for neuroinflammation and BBB dysfunction

S100B, primarily released from astrocytes, serves as a surrogate for BBB disruption and glial activation [25,50]. Its association with hypotension episodes and the presence of encephalopathy in our study suggests that hypotension-induced ischemic BBB injury and secondary neuroinflammation may underlie the observed neurological manifestations. Experimental models show that S100B can both reflect and amplify neuroinflammation through RAGE-dependent pathways, while clinical studies have linked elevated S100B to poor neurological outcomes in primary neurological diseases and sepsis [27,51]. Although S100B is not entirely brain-specific, its correlation with encephalopathy and hypotension supports its use as a pragmatic bedside biomarker of encephalopathy in shock.

### Septic vs. non-septic shock: a convergent phenotype of brain dysfunction

Despite distinct inflammatory and endothelial profiles, septic and non-septic shock patients exhibited similar encephalopathy and brain injury rates. This observation implies that circulatory failure and hypotension-induced BBB injury and neuroinflammation represent a final common pathway of brain injury across shock etiologies. Non-septic shock, particularly cardiogenic shock, often required prolonged vasopressor or mechanical support (IABP/ECMO) and was characterized by more frequent severe hypotension, reinforcing the link between MAP instability and encephalopathy [52,53].

### Individualized cerebral perfusion targets

Our results showed that repeat MAP <50 mmHg may be associated with cerebral hypoperfusion in shock although the relation between MAP and cerebral perfusion is not uniform across patients. Although MAP ≈ 50 mmHg represent the theoretical lower limit of autoregulation, this threshold can shift rightward with chronic hypertension, altered PaCO₂, or vasopressor use [33,34]. Consequently, brief or repeated MAP <50 mmHg episodes may trigger regional ischemia even when global MAP targets are met [3,31,32]. In line with recent work advocating autoregulation-guided “optimal MAP” approaches using transcranial doppler (TCD) or Near-Infrared- Spectroscopy (NIRS), maintaining a fixed MAP threshold is not universally applicable [54,55]. Instead, avoid recurrent severe hypotension and individualizing perfusion MAP targets are likely strategies to mitigate encephalopathy and brain injury.

### Clinical and translational implications

From a clinical standpoint, our findings underscore three points: (1) Repeat severe hypotension (MAP <50 mmHg) may be associated with cerebral hypoperfusion in shock states, recognizing that autoregulation may be impaired. (2) Systemic inflammatory biomarkers alone are unreliable predictors of neurological injury in shock; bedside neuro-monitoring (S100B, electroencephalogram, NIRS) should be considered for early detection. 3. Individualized hemodynamic management, guided by autoregulation monitoring where feasible, may optimize cerebral perfusion and reduce neuroinflammatory risk.

Future prospective studies should integrate continuous cerebral monitoring and multimodal biomarker panels (e.g., S100B, neurofilament light chain, Glial fibrillary acidic protein…) to validate these observations and refine patient-specific perfusion thresholds.

### Limitations

This study has several limitations. First, the retrospective design precludes causal inference and is subject to selection bias. Second, cytokine measurements (interleukines −1/−6, Tumor necrosis factor alpha, interferon…) were not available; we used CRP, MMP-9, ICAM-1, and VEGF as feasible proxies of inflammation and endothelial activation. Third, cerebral autoregulation and microcirculatory flow were not directly measured; although TCD and NIRS can provide qualitative insights, they lack the spatial resolution of MRI or CT perfusion imaging. Fourth, neuroimaging was performed only when clinically indicated, likely underestimating subclinical lesions. Fifth, although septic shock was less prevalent in older than younger patients; age-related distribution reflects cohort selection characteristics rather than a biological or protective effect of age. Finally, S100B is not fully brain-specific, though extracerebral sources were minimized by stringent exclusion criteria.

Despite these limitations, our findings delineate a plausible pathophysiologic link between recurrent hypotension, BBB dysfunction, and encephalopathy, independent of systemic inflammation, offering testable hypotension for future mechanistic and interventional studies.

## Conclusions

Encephalopathy in circulatory shock appears predominantly perfusion-driven, arising from repeated severe hypotension and consequent BBB dysfunction and neuroinflammation rather than systemic inflammatory activation.

## CRediT authorship contribution statement

DNN, LH, HZ conceived and designed the study.

DNN, TMN, MD, DR, HDC collected the data.

JS and JS performed the measurements of biomarkers.

DNN, WC analyzed the data.

DNN and TMN wrote the first draft of the manuscript.

LH, HZ revised the text for intellectual content.

All authors read and approved the final manuscript.

## Consent for publication

No applicable.

## Ethics approval and consent to participate

Not applicable.

## Funding

No external funding.

## Availability of data and materials

The datasets analyzed during the current study are available from the corresponding author on reasonable request.

## Declaration of competing interest

The authors have no competing of interests to declare related to this manuscript.
