# Supplementary material for: Severe hypotension but not systemic inflammation or endothelial activation predicts encephalopathy in circulatory shock
Source: Ann Intensive Care. 2026 Feb 18;16:100033. doi: 10.1016/j.aicoj.2026.100033 (PMC12934433; doi:10.1016/j.aicoj.2026.100033)
Supplement: Supplementary file 1 [file mmc1.docx]

Supplementary Table S1: ICU clinical characteristics, biological at inclusion.

|  | Total  n= 198 | Encephalopathy  at admission  n= 81 (41 %) | Encephalopathy  after sedation  withdrawal  n= 59 (30 %) | p-value |
| --- | --- | --- | --- | --- |
| Characteristics | | | | |
| Age (years) | 71 (61, 78) | 73 (63, 81) | 74 (61, 78) | 0. 421 |
| Females, n (%) | 68 (34) | 25 (31) | 18 (31) | 1.00 |
| Septic shock, n (%) | 95 (48) | 49 (60) | 20 (34) | **<0.01** |
| Glasgow Coma Scale | 15 (12, 15) | 12 (8, 13) | 15 (14, 15) | **<0.01** |
| APACHE scores | 78 (56, 103) | 81 (60, 104) | 74 (56, 96) | 0.108 |
| SOFA score at inclusion | 7 (5, 9) | 7 (5, 9) | 7 (5, 9) | 0.577 |
| Charlson comorbidity index | 5 (3, 6) | 5 (4, 7) | 5 (3, 6) | 0.290 |
| Co-morbidities, n (%) |  |  |  |  |
| Diabetes | 41 (21) | 21 (26) | 12 (20) | 0.268 |
| Smoker and alcohol abuse | 57 (29) | 26 (32) | 15 (26) | 0.850 |
| Chronic pulmonary disorder | 58 (29) | 32 (39) | 13 (22) | 0.081 |
| Arterial hypertension | 109 (55) | 51 (63) | 30 (51) | 0.341 |
| Coronary artery disease | 95 (48) | 45 (55) | 20 (39) | 0.611 |
| History of brain disorders | 57 (29) | 18 (22) | 12 (23) | 0.875 |
| History of sepsis | 46 (23) | 18 (22) | 12 (23) | 0.932 |
| History of kidney injury | 43 (22) | 22 (27) | 11 (21) | 0.612 |
| Clinical and biological parameters at inclusion | | | | |
| Temperature (°C) | 37.1 (36.5, 37.7) | 37.1 (36.7, 37.8) | 37.1 (36.5, 37.6) | 0.310 |
| Heart rate (beats/min) | 106 (92, 121) | 103 (92, 120) | 110 (90, 127) | 0.420 |
| Lowest mean arterial pressure (mmHg) | 65 (57, 73) | 57 (50, 69) | 63 (57, 70) | 0.053 |
| pH lowest | 7.31 (7.24, 7.37) | 7.31 (7.21, 7.36) | 7.32 (7.25, 7.36) | 0.645 |
| PaCO2 (mmHg) | 44 (38, 50) | 45 (40, 54) | 44 (38, 48) | 0.170 |
| PaO2/FiO2 ratio | 157 (100, 222) | 151 (101, 245) | 162 (96, 214) | 0.916 |
| Lactate (mmol/L) | 2 (1, 3.6) | 2 (1.2, 3.5) | 2 (1.3, 3.7) | 0.956 |
| Hemoglobin (g/dl) | 9.4 (8.1, 11.5) | 9.5 (8.2, 11.8) | 9 (7.9, 11.2) | 0.527 |
| Platelets (10.6/L) | 150 (92, 261) | 186 (97, 283) | 140 (86, 223) | 0.106 |
| Urea (mg/dl) | 56 (36, 81) | 65 (40, 98) | 57 (38, 79) | 0.149 |
| Creatinine (mg/dl) | 1.26 (0.92, 1.8) | 1.3 (1, 1.87) | 1.2 (1, 1.7) | 0.539 |
| Dobutamine, n (%) | 109 (53) | 35 (47) | 29 (53) | 0.613 |
| Dobutamine (µg/kg/min) | 3 (0, 5) | 0 (0, 5) | 2 (0, 5) | 0.626 |
| Noradrenaline, n (%) | 119 (60) | 44 (59) | 34 (62) | 0.856 |
| Noradrenaline (µg/kg/min) | 0.07 (0, 0.16) | 0.07 (0, 0.15) | 0.08 (0, 0.22) | 0.548 |
| Remifentanil, n (%) | 154 (77) | 57 (76) | 43 (78) | 0.935 |
| Remifentanil (µg/kg/min) | 0.01 (0, 0.02) | 0.01 (0, 0.05) | 0.01 (0, 0.04) | 0.437 |
| Midazolam, n (%) | 64 (32) | 25 (33) | 17 (31) | 0.918 |
| Midazolam (ml/min) | 1.5 (0, 2.4) | 1.5 (0, 2.5) | 1.3 (0, 2.5) | 0.377 |
| Propofol, n (%) | 155 (78) | 57 (76) | 44 (80) | 0.473 |
| Propofol (ml/min) | 3.6 (1, 4.6) | 3.6 (0.8, 5.1) | 3.6 (1.7, 5.6) | 0.496 |
| Biomarkers values |  |  |  |  |
| Serum biomarkers  (normal values) | **Total**  **n= 198** | **Encephalopathy**  **at admission**  **n= 81 (41%)** | **Encephalopathy**  **after sedation withdrawal**  **n= 59 (30%)** | **p-value** |
| S100B (< 0.105µg/L) | 0.13 (0.08, 0.23) | 0.14 (0.08, 0.26) | 0.13 (0.09, 0.23) | **0.029** |
| MMP-9 (< 21ng/L) | 98 (49, 160) | 101 (62, 159) | 84 (45, 187) | 0.661 |
| C-reactive protein  (< 5mg/dl) | 89 (24, 197) | 106 (39, 224) | 93 (29, 196) | 0.350 |
| ICAM-1 (< 34pg/L) | 311 (226, 520) | 318 (238, 543) | 313 (209, 496) | 0.231 |
| VEGF (< 31pg/L) | 313 (149, 663) | 311 (164, 662) | 332 (128, 664) | 0.219 |
