# Supplementary material for: Severe hypotension but not systemic inflammation or endothelial activation predicts encephalopathy in circulatory shock
Source: Ann Intensive Care. 2026 Feb 18;16:100033. doi: 10.1016/j.aicoj.2026.100033 (PMC12934433; doi:10.1016/j.aicoj.2026.100033)
Supplement: Supplementary file 2 [file mmc2.docx]

Supplementary Table S2: ICU clinical characteristics, biological at inclusion.

|  | Total  n= 198 | Age ≤ 65  n= 68 (34 %) | AGE > 65  n= 130 (66 %) | p-value |
| --- | --- | --- | --- | --- |
| Characteristics | | | | |
| Females, n (%) | 68 (34) | 19 | 49 | 0.224 |
| Septic shock, n (%) | 95 (48) | 41 (60) | 54 (41) | **0.018** |
| APACHE scores | 78 (56, 103) | 74 (54, 92) | 80 (58, 106) | **0.017** |
| SOFA score at inclusion | 7 (5, 9) | 7 (5, 9) | 7 (5, 9) | 0.695 |
| Charlson comorbidity index | 5 (3, 6) | 3 (2, 4) | 5 (4, 6) | **< 0.01** |
| Co-morbidities, n (%) |  |  |  |  |
| Diabetes | 41 (21) | 12 (18) | 29 (22) | 0.559 |
| Smoker and alcohol abuse | 57 (29) | 29 (43) | 28 (24) | **< 0.01** |
| Chronic pulmonary disorder | 58 (29) | 15 (22) | 43 (33) | 0.146 |
| Arterial hypertension | 109 (55) | 28 (41) | 81 (62) | **< 0.01** |
| Coronary artery disease | 95 (48) | 21 (31) | 74 (57) | **< 0.01** |
| History of brain disorders | 57 (29) | 22 (32) | 35 (27) | 0.524 |
| History of sepsis | 46 (23) | 21 (31) | 25 (19) | 0.095 |
| History of kidney injury | 43 (22) | 13 (19) | 30 (27) | 0.645 |
| Clinical and biological parameters at inclusion | | | | |
| Temperature (°C) | 37.1 (36.5, 37.7) | 37.3 (36, 38) | 37 (36.5 ,37.5) | 0.032 |
| Heart rate (beats/min) | 106 (92, 121) | 112 (99, 131) | 106 (91, 117) | 0.347 |
| Lowest mean arterial pressure (mmHg) | 65 (57, 73) | 67 (58, 73) | 64 (57, 73) | 0.186 |
| pH lowest | 7.31 (7.24, 7.37) | 7.3 (7.23, 7.37) | 7.32 (7.25, 7.38) | 0.443 |
| PaCO2 (mmHg) | 44 (38, 50) | 45 (39, 52) | 43 (48, 50) | 0.261 |
| PaO2/FiO2 ratio | 157 (100, 222) | 161 (95, 215) | 157 (108, 225) | 0.851 |
| Lactate (mmol/L) | 2 (1, 3.6) | 2.2 (1.3, 3.4) | 1.8 (1.2, 3.6) | 0.640 |
| Hemoglobin (g/dl) | 9.4 (8.1, 11.5) | 9.45 (8.3, 11.4) | 9.4 (8, 11.5) | 0.527 |
| Platelets (10.6/L) | 150 (92, 261) | 151 (94, 266) | 150 (90, 246) | 0.780 |
| Urea (mg/dl) | 56 (36, 81) | 40 (29, 73) | 63 (42, 89) | **< 0.01** |
| Creatinine (mg/dl) | 1.26 (0.92, 1.8) | 1.6 (0.8, 1.8) | 1.5 (1, 1.8) | 0.187 |
| Dobutamine, n (%) | 109 (53) | 34 (50) | 75 (58) | 0.377 |
| Dobutamine (µg/kg/min) | 3 (0, 5) | 1 (0, 5) | 3 (0, 5) | 0.395 |
| Noradrenaline, n (%) | 119 (60) | 44 (65) | 75 (58) | 0.421 |
| Noradrenaline (µg/kg/min) | 0.07 (0, 0.16) | 0.1 (0, 0.15) | 0.05 (0, 0.17) | 0.394 |
| Remifentanil, n (%) | 154 (77) | 58 (85) | 96 (74) | 0.100 |
| Remifentanil (µg/kg/min) | 0.01 (0, 0.02) | 0 ( 0, 0.01) | 0 (0, 0.04) | 0.437 |
| Midazolam, n (%) | 64 (32) | 46 (33) | 18 (31) | 0.235 |
| Midazolam (ml/min) | 1.5 (0, 2.4) | 1.5 (0, 2) | 1.4 (0, 3) | **0.042** |
| Propofol, n (%) | 155 (78) | 110 (82) | 45 (78) | 0.867 |
| Propofol (ml/min) | 3.6 (1, 4.6) | 3.6 (1, 5.4) | 3.4 (1, 5.9) | 0.653 |
|  |  |  |  |  |
