# Supplementary material for: Severe hypotension but not systemic inflammation or endothelial activation predicts encephalopathy in circulatory shock
Source: Ann Intensive Care. 2026 Feb 18;16:100033. doi: 10.1016/j.aicoj.2026.100033 (PMC12934433; doi:10.1016/j.aicoj.2026.100033)
Supplement: Supplementary file 3 [file mmc3.docx]

Supplementary Table S3: Neurological ICU complications and evolution.

| Neurological ICU complications | | | | Total  n= 198 | | age ≤ 65  n= 68 (34 %) | | AGE > 65  n= 130 (66 %) | p-value |
| --- | --- | --- | --- | --- | --- | --- | --- | --- | --- |
| Glasgow Coma Scale | | | | 15 (12,15) | | 15 (13, 15) | | 15 (12, 15) | 0.148 |
| Encephalopathy, n (%) | | | | 140 (71) | | 41 (60) | | 99 (76) | **0.03** |
| Coma, n (%) | | | | 31 (16) | | 8 (12) | | 25 (19) |  |
| Delirium, n (%) | | | | 99 (51) | | 32 (47) | | 67 (51) |  |
| Delirium, days | | | | 2 (0, 5) | | 2 (0, 3) | | 2 (0, 7) | 0.247 |
| Stroke | | | | 10 (5) | | 1 (1) | | 9 (7) | 0.054 |
| Number of patients underwent Brain CT, n (%) | | | | 77 (39) | | 21 (31) | | 56 (43) |  |
| *Normal* | | | | 23 (30) | | 11 (16) | | 12 (9) | **< 0.01** |
| *Structural brain injury, n (%)* | | | | 54 (27) | | 9 (13) | | 45 (35) | **< 0.01** |
| *Large Stroke, n (%)* | | | | 10 (5) | | 1 (1) | | 9 (7) | 0.054 |
| *Other brain lesions, n (%)* | | | | 44 (27) | | 8 (12) | | 36 (27) | **0.015** |
| *Atrophy, n (%)* | | | | 20 (10) | | 5 (7) | | 15 (11) | 0.152 |
| *Small hemorrhage, n (%)* | | | | 2 (1) | | 1 (1) | | 1(1) | 0.892 |
| *Small ischemia, n (%)* | | | | 22 (11) | | 2 (3) | | 20 (15) | **< 0.01** |
| ICU evolution |  |  |  | |  | |  |  |  |
| Mechanical ventilation, n (%) | | | | 186 (94) | | 64 (94) | | 122 (94) | 0.857 |
| Mechanical ventilation, days | | | | 6 (4, 16) | | 7 (4, 22) | | 6 (3, 15) | 0.146 |
| Sedation, n (%) | | | | 186 (94) | | 64 (94) | | 122 (94) | 0.857 |
| Sedation, days | | | | 5 (3, 10) | | 6 (3, 13) | | 5 (3, 8) | 0.091 |
| Vasopressors, n (%) | | | | 151 (76) | | 51 (75) | | 100 (77) | 0.945 |
| Vasopressors, days | | | | 4 (3, 5) | | 3 (1, 5) | | 3 (1, 6) | 0.591 |
| ECMO or IABP, n (%) | | | | 59 (30) | | 14 (21) | | 45 (35) | **0.041** |
| Episode of mean arterial pressure < 50mmHg over 3 study days, n | | | | 270 | | 47 | | 223 | **< 0.01** |
| Mean arterial pressure < 50mmHg,  duration (minutes) | | | | 5 (0, 30) | | 5 (0, 22) | | 5 (0, 30) | 0.183 |
| Episode of mean arterial pressure < 60mmHg over 3 study days, n | | | | 780 | | 151 | | 639 | **< 0.01** |
| Mean arterial pressure < 60mmHg,  duration (minutes) | | | | 30 (0, 127) | | 30 (0, 85) | | 60 (10, 150) | **0.026** |
| SOFA score at day three | | | | 6 (3, 8) | | 5 (2, 8) | | 6 (3, 8) | 0.054 |
| ICU mortality, n (%) | | | | 71 (36) | | 19 (28) | | 52 (40) | 0.127 |
| ICU stay, days | | | | 13 (7, 23) | | 13 (8, 23) | | 13 (7, 22) | 0.569 |
| In-hospital mortality after ICU, n (%) | | | | 14 (3) | | 1 (1) | | 13 (10) | **< 0.01** |
| In-hospital stay, days | | | | 27 (15, 44) | | 27 (14, 42) | | 27 (15, 45) | 0.873 |
| ICU-acquired infection, n (%) | | | | 102 (51) | | 31 (46) | | 71 (55) | 0.290 |
| Acute kidney injury, n (%) | | | | 72 (36) | | 23 (34) | | 49 (38) | 0.702 |
| Acute Respiratory distress syndrome, n (%) | | | | 20 (10) | | 7 (10) | | 13 (10) | 0.899 |
| Acute heart failure, n (%) | | | | 50 (25) | | 11 (16) | | 39 (30) | **0.033** |
