# Supplementary material for: Severe hypotension but not systemic inflammation or endothelial activation predicts encephalopathy in circulatory shock
Source: Ann Intensive Care. 2026 Feb 18;16:100033. doi: 10.1016/j.aicoj.2026.100033 (PMC12934433; doi:10.1016/j.aicoj.2026.100033)
Supplement: Supplementary file 4 [file mmc4.docx]

| *Day 1* | S100B | CRP | ICAM-1 | VEGF | MMP-9 |
| --- | --- | --- | --- | --- | --- |
| S100B | 1 |  |  |  |  |
| CRP | -0,090 | 1 |  |  |  |
| ICAM-1 | -0,050 | 0,359 ** | 1 |  |  |
| VEGF | -0,088 | 0,441 ** | 0,213 | 1 |  |
| MMP-9 | 0,045 | 0,042 | -0,005 | 0,055 | 1 |
|  | | | | | |
| *Day 2* | **S100B** | **CRP** | **ICAM-1** | **VEGF** | **MMP-9** |
| S100B | 1 |  |  |  |  |
| CRP | -0,037 | 1 |  |  |  |
| ICAM-1 | -0,039 | 0,321 ** | 1 |  |  |
| VEGF | -0,087 | 0,267 ** | 0,174 | 1 |  |
| MMP-9 | 0,017 | 0,030 | 0,004 | 0,017 | 1 |
|  | | | | | |
| *Day 3* | **S100B** | **CRP** | **ICAM-1** | **VEGF** | **MMP-9** |
| S100B | 1 |  |  |  |  |
| CRP | -0,101 | 1 |  |  |  |
| ICAM-1 | -0,038 | 0,155 ** | 1 |  |  |
| VEGF | -0,109 | 0,056 | 0,184 | 1 |  |
| MMP-9 | 0,082 | 0,06 | 0,026 | 0,015 | 1 |

Supplementary table S4.

Correlation between five biomarkers over three days.

** statistically significant with p< 0.05
