# Supplementary figures and images for: Severe hypotension but not systemic inflammation or endothelial activation predicts encephalopathy in circulatory shock
Source: Ann Intensive Care. 2026 Feb 18;16:100033. doi: 10.1016/j.aicoj.2026.100033 (PMC12934433; doi:10.1016/j.aicoj.2026.100033)

## Slide 1
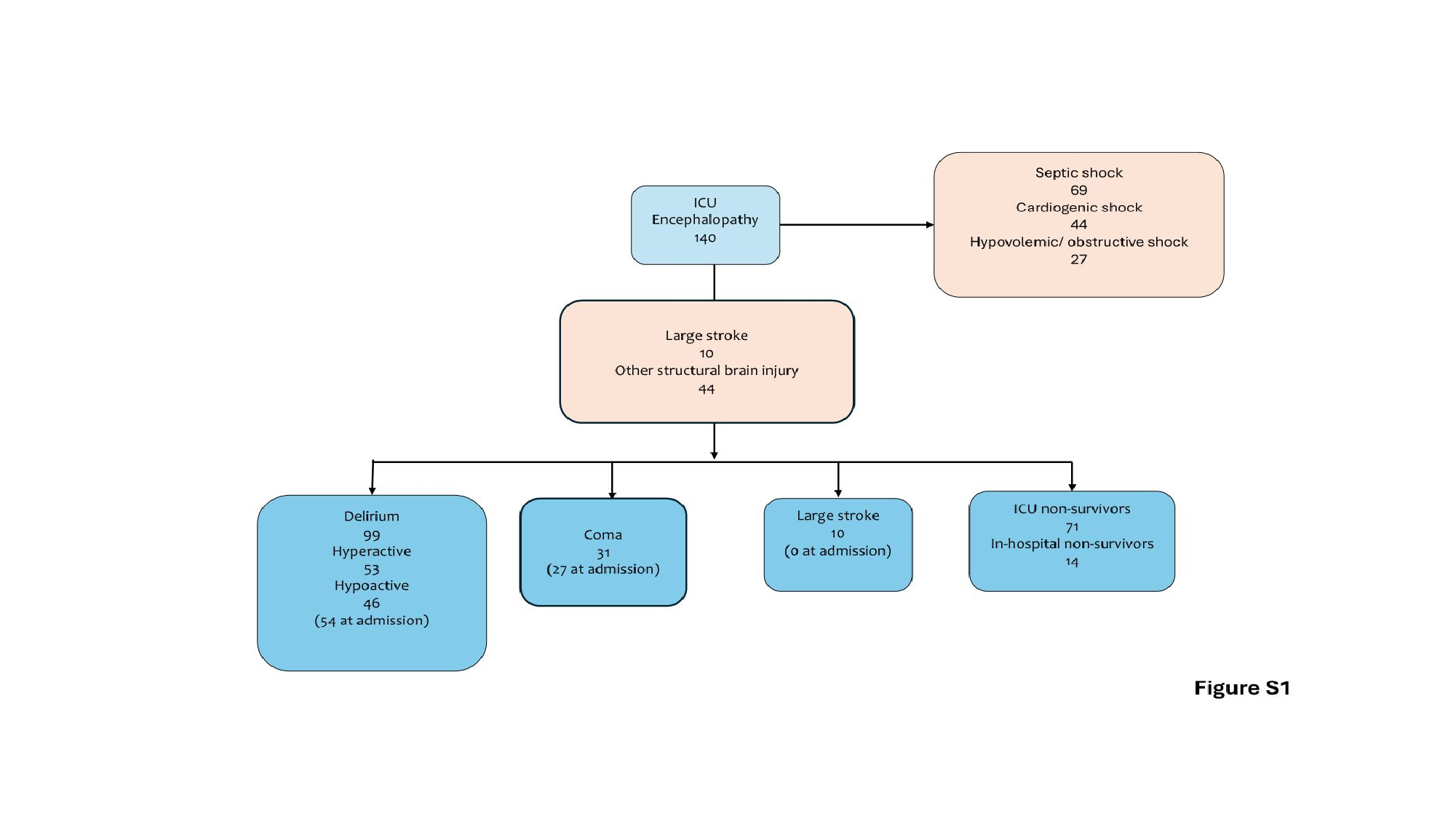

## Slide 2
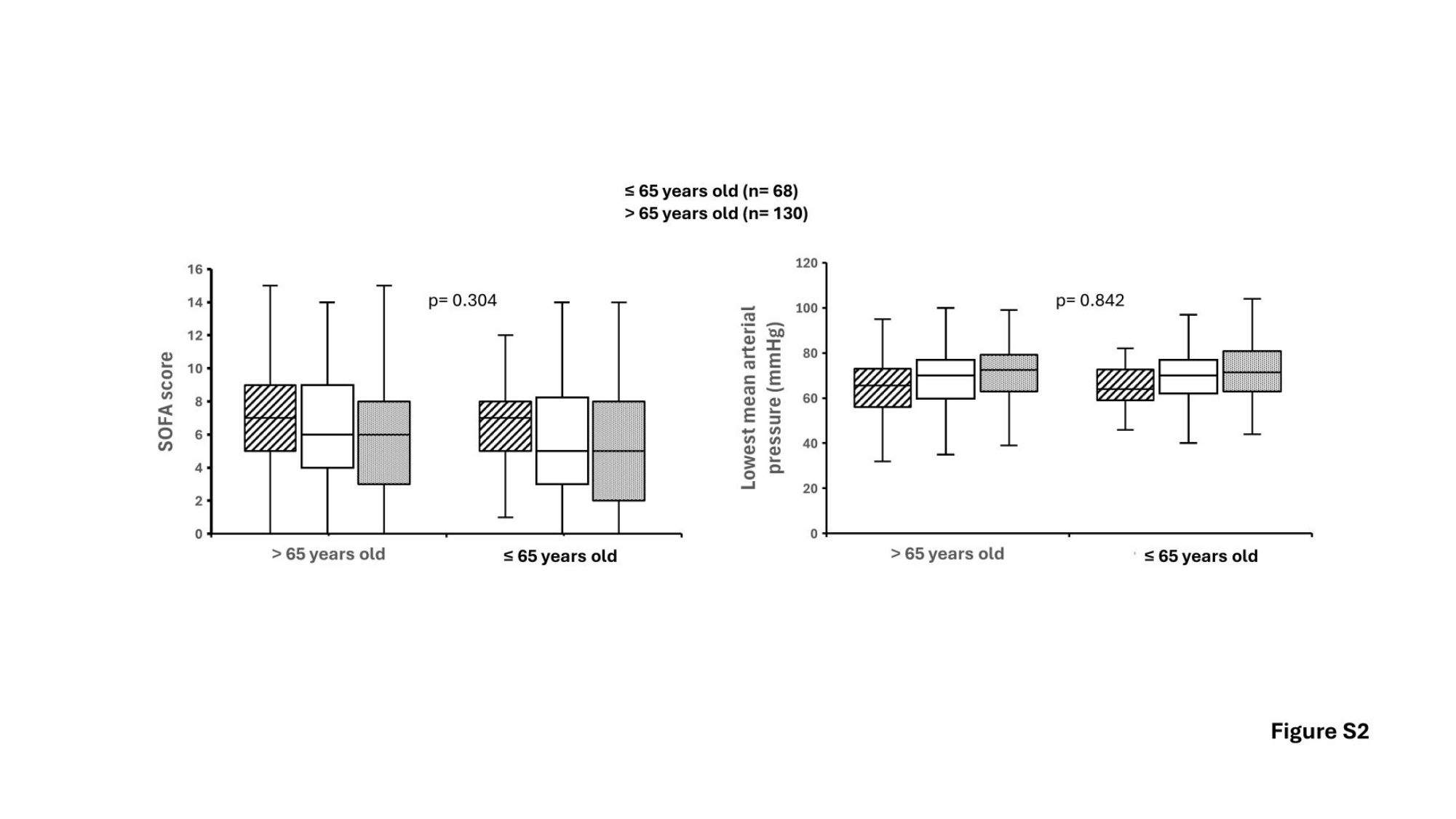

## Slide 3
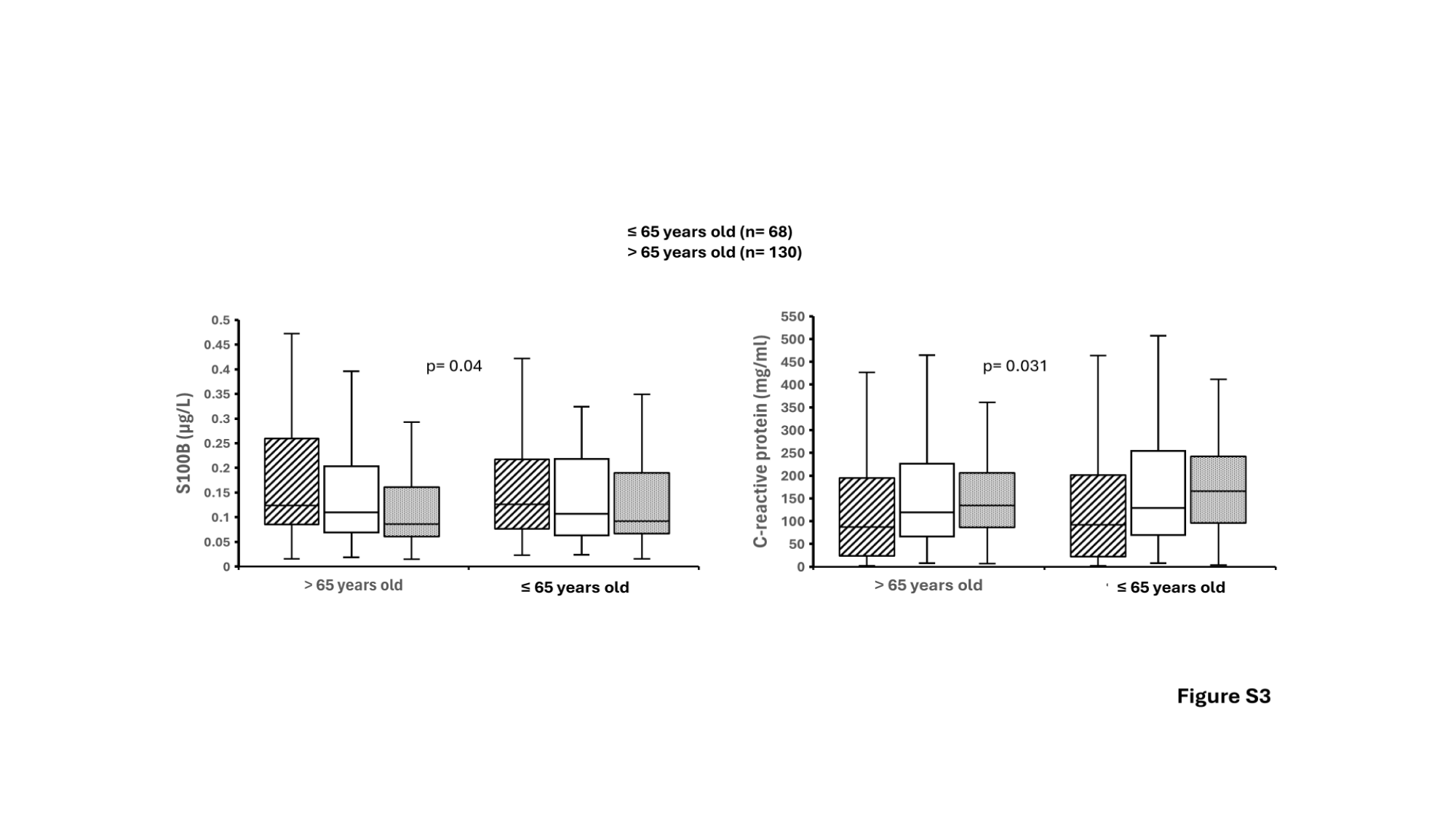

## Slide 4
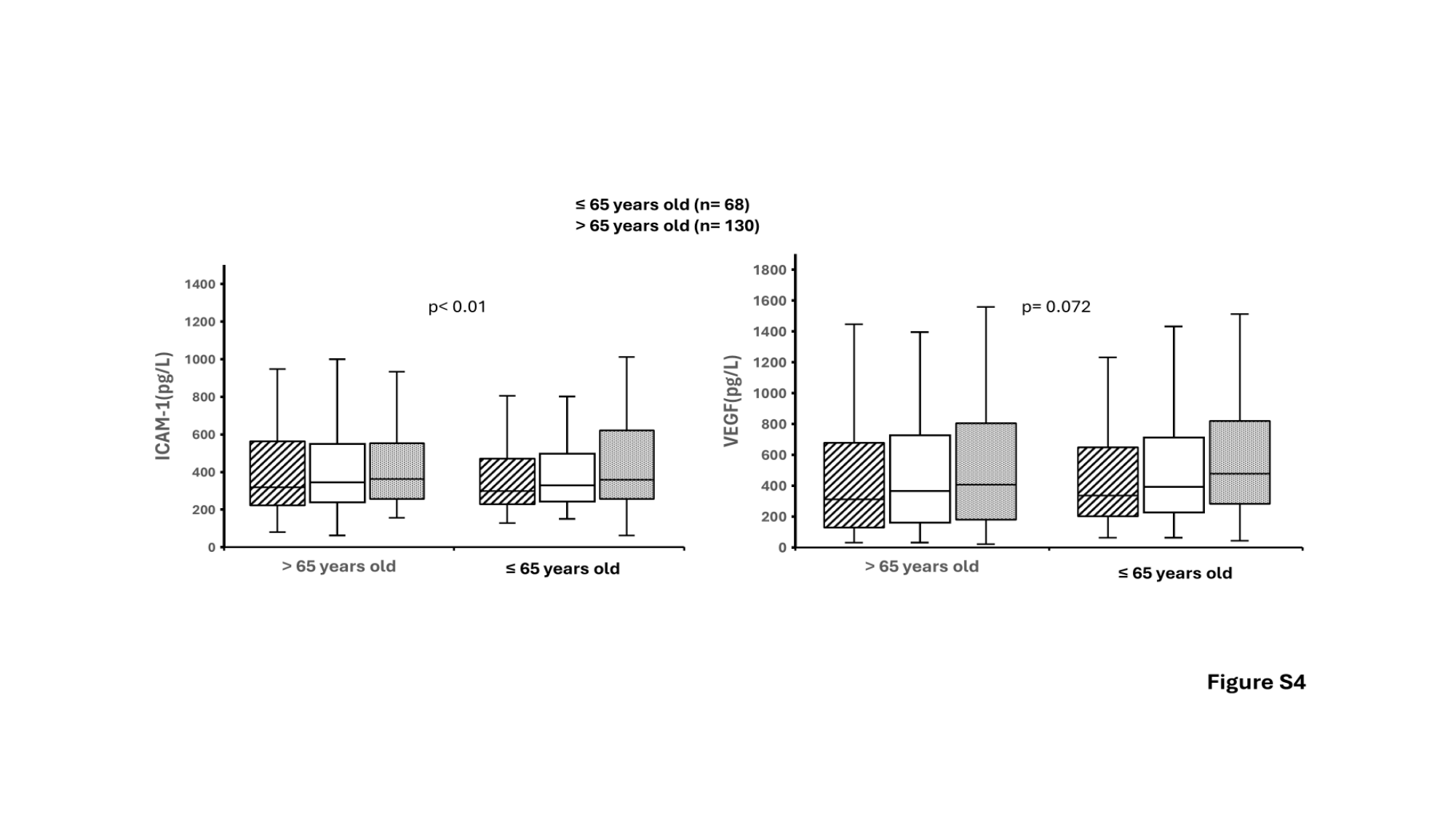

Supplement: Supplementary file 5 [file mmc5.pptx]
